# No Evidence for the Involvement of Cognitive Immunisation in Updating Beliefs About the Self in Three Non-Clinical Samples

**DOI:** 10.1007/s10608-021-10256-y

**Published:** 2021-07-30

**Authors:** Tobias Kube, Julia Anna Glombiewski

**Affiliations:** Pain and Psychotherapy Research Lab, University of Koblenz-Landau, Ostbahnstraße 10, 76829 Landau, Germany

**Keywords:** Belief updating, Cognitive immunisation, Expectation, Depression, Reappraisal

## Abstract

**Background:**

Cognitive immunisation against disconfirmatory evidence (i.e., devaluing expectation-disconfirming information through cognitive mechanisms) has recently been discussed as an obstacle to the revision of dysfunctional beliefs in mental disorders such as depression. Yet, it is unclear whether cognitive immunisation is also involved in belief updating in non-clinical samples.

**Methods:**

Using a three-group modulation protocol (promotion vs. inhibition of cognitive immunisation vs. control group), we examined how cognitive immunisation influences belief updating in response to performance feedback in three non-clinical samples. In Experiments 1 (*N* = 99) and 2 (*N* = 93), participants received unexpectedly negative feedback, whereas participants from Experiment 3 (*N* = 118) received unexpectedly positive feedback. Depressive symptoms and dispositional optimism were examined as additional predictors of belief updating.

**Results:**

In all experiments, participants adjusted their expectations in line with the feedback received, but this effect was not influenced by the cognitive immunisation manipulation. In Experiment 3, expectation change remained stable over 2 weeks. Depressive symptoms were associated with a reduced integration of positive feedback, but not with an increased sensitivity to negative feedback.

**Conclusions:**

Whereas previous research has shown that cognitive immunisation contributes to persistent beliefs in clinical populations, the present findings suggest that it does not affect belief updating in non-clinical samples.

**Supplementary Information:**

The online version contains supplementary material available at 10.1007/s10608-021-10256-y.

## Introduction

Dysfunctional beliefs are core features of almost all mental disorders (Rief et al., 2015). An important question from a clinical point of view is whether such beliefs are revised when novel experiences are made that disconfirm them. The area of research dealing with this phenomenon is the study of belief updating (Kube & Rozenkrantz, 2021; Sharot & Garrett, 2016). It has been assumed that supporting information stabilises prior beliefs, whereas discrepancies between predicted and actual outcomes normally entail an update of beliefs (Barrett & Simmons, 2015; Friston & Kiebel, 2009; Kanai et al., 2015). Yet, with regard to the latter, several lines of research have converged on the finding that the extent to which beliefs are updated after receiving disconfirming information critically depends on the valence of such information (Bromberg-Martin & Sharot, 2020; Sharot & Garrett, 2016). Specifically, Sharot et al. have shown in a well-designed series of experiments that healthy people update their beliefs to a greater extent in response to information that is better than expected (“good news”) than in response to worse-than-expected information (“bad news”) (Garrett & Sharot, 2017; Sharot et al., 2007, 2011, 2012). This asymmetry in belief updating has been referred to as the optimism bias (Sharot, 2011). Relatedly, dispositional optimism—in terms of a relatively stable personality trait (Scheier & Carver, 1985)—has been associated with increased belief updating towards good news, relative to bad news (Sharot et al., 2011).

In clinical populations, the optimism bias has been shown to be absent, e.g., in people with depression (Garrett et al., 2014; Korn et al., 2014), autism spectrum disorder (Kuzmanovic et al., 2019; Rozenkrantz et al., 2021), and borderline personality disorder (Korn et al., 2016). Instead, several mental disorders are related to the persistence of disorder-specific beliefs despite disconfirmatory evidence as indicated in research social anxiety (Koban et al., 2017), obsessive–compulsive disorder (Moritz & Jelinek, 2009; Moritz & Pohl, 2009), psychosis (Buchy et al., 2007; Woodward et al., 2006, 2008), and persistent somatic symptoms (Rief et al., 2006). Most evidence in this regard has been provided by research on depression: In this area of research, it has been shown that depression is related to negative beliefs, and once these beliefs are established, they are maintained despite disconfirming positive information (Everaert et al., 2018, 2020; Kube, et al., 2019a, b, c; Liknaitzky et al., 2017).

To explain the persistence of expectations—representing the subset of beliefs that refer to future events or experiences (Kube et al., 2017; Laferton et al., 2017; Olson et al., 1996)—the concept of cognitive immunisation against disconfirmatory evidence has been introduced. According to this concept, people use cognitive strategies to reduce the discrepancy between their expectations and actual outcomes (Panitz et al., 2021; Rief et al., 2015). Specifically, it has been distinguished between concept-oriented immunisation, referring to a conceptual reframing of disconfirming information, and data-oriented immunisation, relating to a devaluation of disconfirming information. The latter, which will be of particular relevance for the present article, includes strategies such as assigning low reliability or credibility to discrepant information (Dunn & Schweitzer, 2005; Sarathchandra & Haltinner, 2020) and subtyping, i.e., categorising the outcome as an exception to the rule (Bless et al., 2001; Carnaghi & Yzerbyt, 2007). In other words, cognitive immunisation means that disconfirmatory evidence is “explained away” such that new information is no longer conflicting with people’s prior expectations. Thus, cognitive immunisation can be regarded as a form of “motivated reasoning” (Kunda, 1990), where people use “defensive” strategies to reduce the dissonance between prior beliefs and conflicting new information (Festinger, 1962).

In clinical psychology, especially in research on depression, it has been shown that promoting the use of cognitive immunisation strategies, e.g., by questioning the reliability of disconfirming information, leads to a reduced update of negative performance-related expectations in response to unexpectedly positive performance feedback; in contrast, inhibiting the use of cognitive immunisation strategies against novel positive information by increasing the assigned value of disconfirming information has been shown to enhance the update of negative expectations (Kube et al., 2019a and c). Healthy people, on the other hand, seem to be less susceptible to devaluing good news through cognitive immunisation strategies, another experiment suggested: In that study, healthy people updated their expectations in line with positive performance feedback received for their performance, regardless of a manipulation designed to question the validity of the feedback (Kube & Glombiewski, 2021). Yet, it is unclear whether the systematic modulation of cognitive immunisation also influences the adjustment of expectations in non-clinical samples, and whether this may depend on the valence of new information. Insights into these questions would be valuable not only with regard to the question of whether the engagement in cognitive immunisation is specific to certain mental disorders (vs. are also pertinent in non-clinical samples), but also as it would shed light on the specific cognitive mechanisms underlying the well-known optimism bias in non-clinical samples (Sharot, 2011).

### Overview of the Present Studies and Hypotheses

We performed three consecutive experiments to examine whether cognitive immunisation against new information is a mechanism underlying the lack of expectation adjustment and whether this depends on the direction of the expectation disconfirmation, i.e., whether the outcome is better than expected or worse than expected. Specifically, with reference to the optimism bias (Sharot, 2011), we hypothesised that if participants receive new information that is worse than expected (i.e. bad news), the promotion of cognitive immunisation (e.g., through questioning the validity of new information) will lead to the maintenance of the initial expectation. On the other hand, if cognitive immunisation is inhibited (e.g., through underscoring the validity of new information), bad news is expected to have more influence on the formation of the posterior expectation, i.e., initiate greater expectation updating. That was the purpose of “Experiment 1” and “Experiment 2”. Conversely, if participants receive information that is better than expected, as performed in “Experiment 3”, the inhibition of cognitive immunisation is supposed to enhance expectation updating in line with positive information, whereas the promotion of cognitive immunisation is expected to block expectation updating. In other words, we predicted that the inhibition of cognitive immunisation is related to increased updating in line with the valence of new information, whereas the promotion of cognitive immunisation was expected to be associated with reduced updating in either case.

In all three experiments, we tested these hypotheses by using a well-established belief-updating task, where the extent to which participants update their performance-related expectations in response to unexpectedly positive vs. negative performance feedback is examined (Kube et al., 2018). In addition to the hypothesised group differences regarding the adjustment of prior expectations, we also examined the influence of depressive symptoms on expectation updating, since previous research has linked depressive symptoms to reduced updating in line with positive feedback (Kube & Glombiewski, 2021; Kube, et al., 2019a, 2019b, 2019c), whereas the influence of depressive symptoms on the adjustment of expectations in response to negative information is less clear (Everaert et al., 2018; Kube, et al., 2019a, b, c). Moreover, since previous research has linked high trait optimism to asymmetric belief updating in response to good news, but not bad news (Sharot et al., 2011), we expected high dispositional optimism to be associated with a reduced update in line with negative feedback (Experiments 1 and 2) and an increased update in response to positive feedback (Experiment 3). Due to a lack of power, we decided not to examine potential moderating effects of depression and/or optimism on the effects of condition.

## General Method

All experiments were approved by the local ethics committee (reference number 2019_195) and were conducted in accordance with ethical standards as laid down in the 1964 Declaration of Helsinki and its later amendments. All participants gave written informed consent and were treated in accordance with the ethical guidelines of the German Psychological Society. All experiments were pre-registered at AsPredicted.org: see section “Experiment 1” and “Experiment 2”: https://aspredicted.org/th9y4.pdf; see section “Experiment 3”: https://aspredicted.org/ht9fj.pdf.

## Experiment 1

### Methods

#### Participants

The sample size was determined via a-priori power analysis based on previous studies using cognitive immunisation manipulations in the context of unexpected performance feedback (Kube et al., 2019a and c). Expecting a small to medium effect, the power analysis (expected *f* = 0.165; power = 0.80; *α* = 0.05; three groups; two measurements; correlation between the measurements: *r* = 0.5) indicated a required sample size of at least 93 participants. Participants were recruited via email lists and postings at the University Campus of Landau. Inclusion criteria were: at least 18 years old and sufficient German language knowledge. Exclusion criterion was the participation in previous studies of our research group on expectation update. We recruited *N* = 102 participants; this slight surplus would allow us to exclude participant data if necessary due to experimental or statistical issues without substantially losing power. Participants received course credit for their participation or, alternatively, had the chance to win gift vouchers for a popular book shop.

### Procedure

**Fig. 1:**
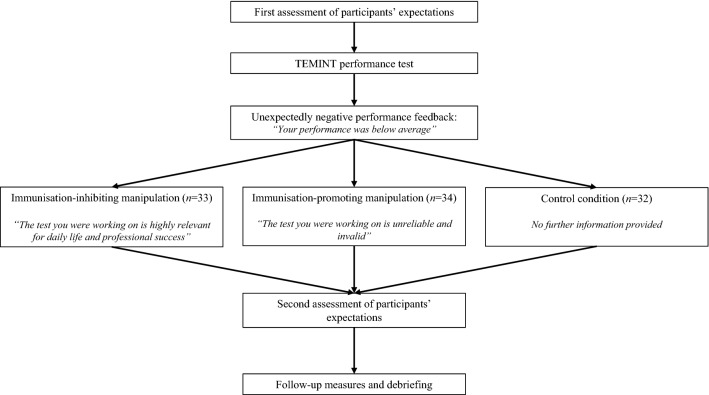
Design of “Experiment 1”

The present study was based on a paradigm developed and validated in previous work (Kube et al., 2018). The experimental sessions of the present study were conducted by a female psychology master student. Data were collected in October and November 2019. All measures were completed in German language online via the commercial survey platform Unipark®. Figure 1 illustrates the study design.

#### Cover Story

To conceal the actual purpose of the study and prevent demand effects, participants were led to believe that the study was about the relationship between current mood and performance. At the beginning of the experimental session, participants were therefore asked to rate their current mood. Then, participants were informed that they would work on a performance test, with which they should not be familiar yet.

#### Performance Test

After rating their expectations for their performance in the upcoming test, participants completed the **T**est of **EM**otional **INT**elligence (TEMINT) (Schmidt-Atzert & Buehner, 2002). This test was used in all previous studies on expectation update in the context of disconfirmatory performance feedback. It was selected primarily because in this test it is difficult for participants to evaluate their own performance, which is important for the performance feedback received to appear credible. The test comprises a total of 12 brief descriptions of situations with one acting person who actually experienced the given situation (e.g. “I had a dispute with a colleague”). Participants are provided with these situations and are asked to empathise with the acting person and to evaluate the degree to which the acting person experienced certain emotions in the scenario (such as anger, fear, happiness). The TEMINT sum score reflects the overall deviations from the actual ratings of the persons mentioned in the situations, with low sum scores indicating good performance in the test. The TEMINT has shown good psychometric properties in previous studies (Blickle et al., 2011; Schmidt-Atzert & Buehner, 2002). Internal consistency of the TEMINT in the present study was *α* = 0.77.

#### Performance Feedback

After each of three blocks of the TEMINT, participants received standardised performance feedback suggesting that their performance was below average. Specifically, participants were informed that they solved only about half of the tasks correctly and that their performance was thus below average in comparison to all previous participants who worked on the test. It was assumed that this feedback would be perceived by participants as being unexpectedly negative, since previous research has shown that (healthy) people tend to consider their abilities usually to be above average (Brookings & Serratelli, 2006; Schwert et al., 2018; Taylor et al., 2000). Feedback was provided after each of three blocks as well as after the entire test. Of note, we did not ask participants to evaluate their performance since previous research has shown that participants’ self-evaluation of their performance in the TEMINT is totally unrelated to their actual performance (Kube et al., 2021).

#### Experimental Conditions

Participants were randomly assigned to one of three conditions: immunisation inhibition; immunisation promotion; or control group. After receiving feedback for the entire test, participants from the immunisation inhibition group were presented an information text on the screen, in which further information on the TEMINT was provided. Specifically, participants from this group were informed that the TEMINT has been shown to be highly reliable and valid performance test, which is relevant for both daily life and professional success. They were told that previous research had found that people performing well on the TEMINT have more professional success, measurable on both subjective (e.g. work satisfaction) and objective measures (e.g., higher income). Furthermore, participants were informed that persons who perform well on the TEMINT are more satisfied with their social lives, including the quality of social relationships. This information text was used to manipulate participants’ appraisal of the feedback received by increasing the value of the unexpected performance feedback. In previous studies, it has been shown that the same manipulation as used in the present studies facilitated the update of negative expectations in response to positive performance feedback in people with depression (Kube et al., 2019a and c). Here, we used this manipulation to examine whether such an evaluation of the feedback received affects the adjustment of expectations in response to negative feedback. We anticipated that after receiving this fake information about the TEMINT, it would be difficult for participants to engage in cognitive immunisation strategies (that is, devaluing the feedback received), because the validity and importance of the feedback received were explicitly highlighted. Thus, we hypothesised that emphasising the validity of the negative test experience would lead participants from the immunisation inhibition condition to update their performance expectations in line with the negative feedback.

The immunisation promotion group received an information text that was similar to the immunisation inhibition group in terms of length and style, but aimed at lowering the value assigned to the performance feedback. Participants from the immunisation promotion group were informed that the TEMINT has been found to be a fairly unreliable and invalid performance test. They were informed that the TEMINT has neither been found to predict professional success nor other aspects of life satisfaction, covering the same aspects as mentioned in the immunisation inhibition condition. We anticipated that after being provided with this information about the TEMINT, it would be easy for participants to engage in cognitive immunisation against the negative feedback received because the validity of the expectation-disconfirming experience was explicitly questioned. Thus, we expected that participants from this condition would show an increased propensity to disregard the negative feedback and maintain their initial performance expectations (hence showing reduced expectation update in comparison with the immunisation inhibition group).

The control group received no further information after completing the test and receiving feedback for their performance.

#### Additional Measures and Debriefing

After completing the post-assessment of participants’ expectations and measures of cognitive immunisation, participants were asked to complete some additional questionnaires including sociodemographic variables, depressive symptoms, and dispositional optimism. Finally, participants were debriefed with respect to the actual purpose of the study.

### Measures

#### Changes in Expectations

Participants’ performance expectations were assessed with the Performance Expectations Scale developed by Kube et al., (2018). This scale comprises both participants’ task-specific expectations (tied to the expected performance in a particular test) and generalised performance expectations (referring to the expected performance in unknown tests in general), each with two items before and two items after working on the test. These two subscales are analysed separately. Half of the items express positive expectations (e.g., “Solving the tasks from the test will be easy for me”), whereas the other half reflect negative expectations (e.g., “Solving unknown tasks in general will be difficult for me”), requiring reverse scores. All items are rated on a seven-point Likert scale ranging from (1) “I totally disagree” to (7) “I totally agree”, so higher values in the sum scores reflect positive performance expectations. Participants rated the Performance Expectations Scale twice: before working on the TEMINT and after the cognitive immunisation manipulation (for the immunisation-promoting and the immunisation-inhibiting condition, respectively). The Performance Expectations Scale has been used in several previous studies and has shown good psychometric properties (Kube et al., 2018, 2019a, b, c). In the present study, Cronbach’s alpha of the task-specific expectations subscale was *α* = 0.77, and Cronbach’s alpha of the generalised performance expectations subscale was *α* = 0.87. As in previous studies (Kube et al., 2019a, b, c), pre to post changes in generalised performance expectations were pre-defined as the primary outcome, whereas the update of task-specific expectations was considered the secondary outcome.

#### Cognitive Immunisation

To assess the degree to which participants questioned or disregarded the negative performance feedback received, we used the Cognitive Immunisation after Performance Feedback (CIPF) scale (Kube et al., 2019a, 2019b, 2019c). Two items assess the extent to which participants appraise the test as being concerned with an important area of their lives; two items assess whether participants consider the feedback received to be credible; and two items assess whether participants tend to disregard the feedback by considering it as an exception. In previous studies using clinical and non-clinical samples, this scale was found to have good psychometric properties and was associated with the update of performance expectations after feedback (Kube et al., 2019a andb). Each item was rated on a Likert scale ranging from (1) “I totally disagree” to (7) “I totally agree”, and higher values reflect a greater engagement in cognitive immunisation strategies. Some of the items were reversely scored, e.g., the items relating to the assessment of the credibility of the feedback, when computing the CIPF sum score. If the cognitive immunisation manipulation was successful, the immunisation-promotion condition should have the highest CIPF sum scores, followed by the control group, while the immunisation-inhibition group was supposed to have the lowest CIPF sum scores. Cronbach’s alpha of the CIPF scale was *α* = 0.66.

#### Depressive Symptoms

To assess depressive symptoms, we used the second edition of the Beck Depression Inventory (BDI- II; (Beck et al., 1996), which comprises 21 items (each rated on a 4-point scale ranging from 0 to 3). The sum score ranges between 0 and 63, with lower values indicating fewer depressive symptoms. In our sample, the internal consistency of the BDI-II was *α* = 0.87.

#### Dispositional Optimism

Dispositional optimism was assessed with the German version of the Life Orientation Test Revised (LOT-R) (Glaesmer et al., 2008). The LOT-R comprises 10 items, four of which are distractor items. All items are rated on a five-point Likert scale ranging from (1) “I totally disagree” to (7) “I totally agree”. High values of the sum score of the LOT-R reflect a more optimistic view of one’s future. The internal consistency of the LOT-R in the present study was *α* = 0.76.

#### Mood

Participants’ current mood was assessed with the Positive and Negative Affect Scale (PANAS) by Watson et al. (1988) Watson et al. (1988). The PANAS is a brief measure that has been used to assess positive and negative emotions independently. The PANAS comprises twenty adjectives, ten of which describing positive emotional states (e.g., excited, proud, and inspired) and ten describing negative emotional states (e.g., distressed, jittery, and upset). Participants noted the extent to which they experienced each emotion in the current situation, using a numerical scale ranging from 0 (not at all) to 10 (extremely strong). The PANAS has shown very good psychometric properties in previous studies (Crawford & Henry, 2004; Terracciano et al., 2003; Watson et al., 1988). In our sample, Cronbach’s alpha of the positive affect (PA) subscale was *α* = 0.86; for the negative affect (NA) subscale it was *α* = 0.81.

#### Socio-demographics

Socio-demographic variables, including age, sex, education, and employment status, were assessed using a brief self-report questionnaire.

## Statistical Analyses

We conducted data screening according to the recommendations of Tabachnick and Fidell (2014) and tested the assumptions of analyses of variance (ANOVA). In terms of a manipulation check, we performed a one-way ANOVA to examine differences between the groups in their endorsement of cognitive immunisation strategies (as assessed with the CIPF total score). For the main analysis, we conducted a 2 (Time: before feedback vs. after feedback) × 3 (Condition: immunisation inhibition vs. immunisation promotion vs. control group) mixed ANOVA, with the generalised performance expectations as the dependent variable. The analysis of most interest is the Time by Condition interaction. To further explore significant group difference as indicated in the ANOVA, paired samples *t*-tests were performed. Finally, we examined whether changes in generalised performance expectations were predicted by depressive symptoms and dispositional optimism across conditions. For this purpose, we performed a multiple linear hierarchical regression analysis, entering the experimental condition as a predictor in the first block, depressive symptoms in the second block, and dispositional optimism in the third block.^1^ The pre to post change scores in generalised expectations were defined as the criterion. For all analyses, type-1 error levels were set at 5% (two-tailed). We present 95% confidence intervals (CI) for the effect sizes, that is, Cohen’s *d* and *ɳ*^2^_*p.*_ All analyses were conducted using IBM SPSS Statistics Version 25.

## Results

### Sample Characteristics

As noted in the pre-registration, we planned to exclude participants from the analyses if one of the following criteria was met: (1) > 3 SD above/below the mean on the dependent variable; (2) expressing serious doubts about the cover story and guessing the real purpose of the study; (3) discontinuing participation in the study before entering 2/3 of all data points. The second criterion applied to three participants (one person from the immunisation-inhibition group; two persons from the control group); the other criteria did not apply to any of the participants. Thus, all analyses were based on a sample of 99 people (*n* = 33 in the immunisation-inhibition condition; *n* = 34 in the immunisation-promotion condition; *n* = 32 in the control group). Participants’ mean age was 22.39 years (*SD* = 5.52). In our sample, 79.8% of the participants were female, and 91.9% were undergraduate students (most of whom studied psychology). Sociodemographic characteristics for the three experimental groups separately can be found in Table 1. BDI-II sum scores (*M* = 9.84; *SD* = 5.52) indicate that on average participants reported minimal levels of depressive symptoms. Seven participants (7.1%) reported elevated levels of depression (BDI-II sum scores ≥ 19).

**Table 1:** Sample characteristics in study 1

Variable	Immunisation-inhibiting group (*n* = 33)	Immunisation-promoting group (*n* = 34)	Control group (*n* = 32)
Age in years, *M* (SD)	21.52 (3.15)	22.12 (6.09)	23.59 (6.66)
Sex, N (%)			
Male	8 (24.2)	6 (17.6)	6 (18.8)
Female	25 (75.8)	28 (82.4)	26 (81.2)
Educational level, N (%)			
High school degree	32 (97.0)	32 (94.1)	32 (100.0)
University degree	1 (3.0)	2 (5.9)	0
Employment status, N (%)			
Full-time working	1 (3.0)	2 (5.9)	2 (6.3)
Part-time working	1 (3.0)	0 (5.1)	1 (3.1)
In training	30 (91.0)	32 (94.1)	29 (90.6)
Unemployed	1 (3.0)	0	0

*M* mean, *SD* standard deviation, *N* number

### Baseline Differences Between Conditions

Participants from the three groups did not differ on initial task-specific expectations, *F* (2, 96) = 1.919, *p* = 0.152, *ɳ*^2^_*p*_ = 0.038, 95% CI [0, 0.124]; initial generalised expectations, *F* (2, 96) = 1.790, *p* = 0.172, *ɳ*^2^_*p*_ = 0.036, 95% CI [0, 0.120]; and age, *F* (2, 96) = 1.220, *p* = 0.300, *ɳ*^2^_*p*_ = 0.025, 95% CI [0, 0.100]. The distribution of male and female participants was not significantly different across the groups, *χ*^2^ = 0.514, *p* = 0.773, nor was the distribution of educational level, *χ*^2^ = 8.223, *p* = 0.083, and employment status, *χ*^2^ = 3.479, *p* = 0.747. Participants from the three groups did not differ in their TEMINT performance either, *F* (2, 99) = 0.441, *p* = 0.645, *ɳ*^2^_*p*_ = 0.009, 95% CI [0, 0.060].

### Manipulation Check

The one-way ANOVA indicated significant differences between the groups in their CIPF total scores, *F* (2, 96) = 3.503, *p* = 0.034, *ɳ*^2^_*p*_ = 0.068, 95% CI [0.001, 0.169]. Pairwise *t*-tests signified that the immunisation-promoting condition had significantly higher CIPF total scores (*M* = 27.56; *SD* = 4.59) than the immunisation-inhibiting condition (*M* = 24.45; *SD* = 5.28), *t* (65) = −2.571; *p* = 0.012; *d* = 0.629, 95% CI [0.135, 1.117], reflecting a medium effect according to Cohen (1988). With respect to the difference between the immunisation-promotion condition and the control group (*M* = 25.50; *SD* = 4.78), the former had somewhat higher CIPF total scores, but this group difference did not reach significance, *t* (64) = 1.786; *p* = 0.079; *d* = 0.440, 95% CI [−0.050, 0.927]. The immunisation-inhibiting condition did not differ from the control group in their CIPF total scores, *t* (63) = −0.836; *p* = 0.406; *d* = 0.208, 95% CI [−0.281, 0.694].

### Main Analyses

#### Changes in Generalised Expectations

The descriptive values for the expectation ratings, presented in Table 2, show that participants’ expectations were relatively optimistic at baseline and declined to be neutral after feedback. Descriptively, the order of the three groups with respect to the update of generalised performance expectations was in the hypothesised direction: cognitive immunisation-inhibiting group > control group > cognitive immunisation-promoting group.

**Table 2:** Descriptive values for the expectation ratings in study 1

Variable	Immunisation-inhibiting group (*n* = 33)	Immunisation-promoting group (*n* = 34)	Control group (*n* = 32)
Task-specific expectations, *M* (*SD*)			
Pre	10.21 (1.76)	10.88 (2.14)	9.94 (2.14)
Post	8.09 (2.35)	9.35 (2.58)	7.34 (2.27)
Generalised expectations, *M* (*SD*)			
Pre	9.64 (2.16)	10.24 (1.84)	9.25 (2.38)
Post	8.30 (2.33)	9.56 (1.96)	8.44 (2.70)

*M* mean, *SD* standard deviation

**Fig. 2:**
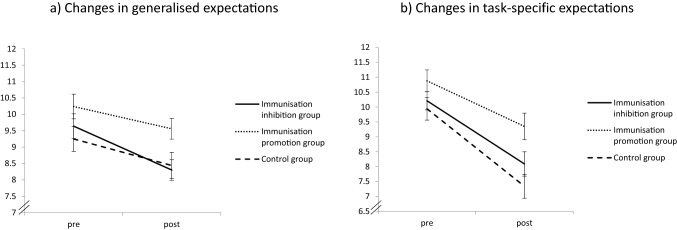
Results for the main analyses from Experiment 1. There were no significant differences between the groups in updating their expectations from pre to post. Error bars reflect the standard error or the mean

In terms of inferential statistics, the Time by Condition two-factorial ANOVA with generalised expectations as the dependent variable indicated a significant main effect of Time, *F* (1, 96) = 17.899, *p* < 0.001, *ɳ*^2^_*p*_ = 0.157, 95% CI [0.046, 0.286]. Overall, participants lowered their expectations from pre (*M* = 9.72, *SD* = 2.15) to post (*M* = 8.78, *SD* = 2.38). The main effect of condition was not significant, *F* (2, 96) = 2.915, *p* = 0.059, *ɳ*^2^_*p*_ = 0.057, 95% CI [0, 0.154]. The Time by Condition interaction was not significant either, *F* (2, 96) = 0.816, *p* = 0.445, *ɳ*^2^_*p*_ = 0.017, 95% CI [0, 0.082].^2^ The adjustment of generalised expectations in the three groups is depicted in Fig. 2a.

#### Changes in Task-specific Expectations

The Time by Condition two-factorial ANOVA with task-specific expectations as the dependent variable indicated a significant main effect of Time, *F* (1, 96) = 45.136, *p* < 0.001, *ɳ*^2^_*p*_ = 0.320, 95% CI [0.173, 0.446], with overall less positive expectations after feedback (*M* = 8.28, *SD* = 2.52) than before feedback (*M* = 10.35, *SD* = 2.04). The main effect of Condition was significant, *F* (2, 96) = 7.274, *p* = 0.001, *ɳ*^2^_*p*_ = 0.132, 95% CI [0.024, 0.249]. Pairwise *t*-tests indicated that, overall, participants from the immunisation-promoting group (*M* = 20.24; *SD* = 3.42) reported significantly more positive expectations than participants from the immunisation-inhibiting group (*M* = 18.30; *SD* = 2.35), *t* (65) = −2.687; *p* = 0.009; *d* = 0.658, 95% CI [0.162, 1.146], and the control group (*M* = 17.28; *SD* = 3.69), *t* (64) = 3.374; *p* = 0.001; *d* = 0.830, 95% CI [0.324, 1.332], reflecting medium to large effects. The difference between the immunisation-inhibiting group and the control group was not significant, *t* (63) = 1.326; *p* = 0.190; *d* = 0.330, 95% CI [−0.1620, 0.817]. As for generalised expectations, the Time by Condition interaction was not significant, *F* (2, 96) = 0.990, *p* = 0.375, *ɳ*^2^_*p*_ = 0.020, 95% CI [0, 0.090]. The descriptive values of the ratings for task-specific expectations are presented in Table 2 and Fig. 2b displays the update of task-specific expectations in the three groups.

#### Considering Negatively Worded Expectations Only

**Fig. 3:**
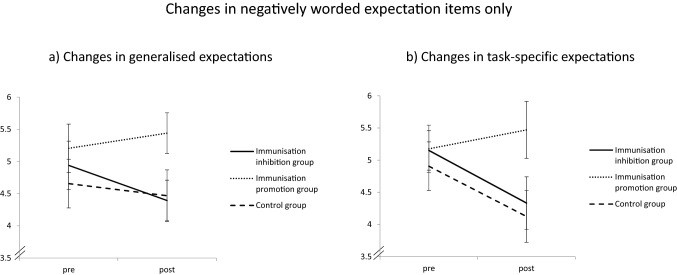
Results from Experiment 1 for expectation change when considering only the negatively worded expectation items (e.g., “Solving the tasks from the test will be difficult for me”). For generalised expectations, participants from the cognitive immunisation inhibition group updated their expectations in a negative direction, whereas participants from the cognitive immunisation promotion group did not. For task-specific expectations, it was found that participants from the cognitive immunisation promotion group updated their expectations to a lesser extent than participants from the cognitive immunisation inhibition group and the control group. Error bars reflect the standard error or the mean

In post-hoc exploratory analyses, we examined whether the pattern of results changes when considering only the negatively worded items of the Performance Expectations Scale. We did so because previous research has shown that (lack of) positive expectation and negative expectations have differential effects on depressive symptoms (Horwitz et al., 2017). Indeed, we found that when including only the negatively worded items in the main analysis, the Time by Condition interaction was more pronounced for the update of both generalised [*F* (2, 96) = 2.560, *p* = 0.056, *ɳ*^2^_*p*_ = 0.058, 95% CI (0, 0.143)] and task-specific expectations [*F*(2, 96) = 3.653, *p* = 0.030, *ɳ*^2^_*p*_ = 0.071, 95% CI (0.001, 0.173)]. However, only the latter reached significance. In particular, participants from the immunisation-inhibiting and control condition lowered their expectations after the negative feedback, whereas participants from the immunisation-promoting condition updated their expectations in a slightly positive direction, as can be seen in Fig. 3. With respect to the update of task-specific expectations, both the immunisation-inhibition condition [*t* (65) = −2.192, *p* = 0.032, *d* = 0.536, 95% CI (0.046, 0.1021)] and the control group [*t* (58.698) = 2.316, *p* = 0.024, *d* = 0.567, 95% CI (0.074, 1.053)] were different from the immunisation-promotion group, post-hoc tests revealed. The immunisation-inhibition group and the control group did not differ from each other, *t* (63) = −0.086, *p* = 0.932, *d* = 0.021, 95% CI [−0.465, 0.508].

### Depressive Symptoms and Trait Optimism as Predictors of Expectation Update

Performing a linear regression analysis, we examined whether depressive symptoms and dispositional optimism were associated with pre to post changes in generalised performance expectations in response to the negative feedback received. Entering the experimental condition in the first block explained 0.9% of the variance, which was not significant (*β* = 0.097; *p* = 0.340). Adding BDI-II sum scores as predictors in the second block explained another 3.7% of the variance, which failed to reach significance (*β* = −0.203; *p* = 0.054). Adding LOT-R sum scores as predictors in the second block did not significantly add explained variance (*ΔR*^2^ = 0.005; *β* = −0.157; *p* = 0.482).

## Discussion

This experiment aimed to examine whether cognitive immunisation against unexpectedly negative performance feedback contributes to optimistic belief updating in a non-clinical sample. The results of the manipulation check showed that the experimental instructions were effective in terms of their differential effects on the appraisal of the negative performance feedback received: As hypothesised, participants from the cognitive immunisation promotion group showed the greatest disregard for the negative feedback on their performance. Yet, the only significant group difference was the difference between the immunisation-promoting and the immunisation-inhibiting condition, and that effect was only medium-sized. With respect to the influence of the cognitive immunisation manipulations on expectation update, we found—contrary to our hypothesis—no significant Time by Condition interaction, meaning that the three groups did not differ in updating their expectations from pre to post. The main effect of time indicated that, overall, participants updated their expectations in line with the negative feedback received; that is, they lowered their expectations from pre to post. One possible interpretation of the lack of group differences in expectation updating is that cognitive immunisation in fact has no influence on the update of generalised performance expectations in response to negative performance feedback. Alternatively, it is possible that the cognitive immunisation manipulation used in the present study was not strong enough, since group differences revealed in the manipulation check were only moderate and the order of the groups in terms of their amount of expectation change was in the hypothesised direction.

An unexpected and hard to interpret finding is that for the update of task-specific expectations, the main effect of condition was significant, whereas the time by condition interaction was not. Possibly, the significant main effect of time relates, at least to some extent, to the slight yet non-significant baseline differences in initial task-specific expectations (*p* = 0.152). Due to randomisation, however, it should be impossible for conditions to have a true effect on participants’ initial expectations; instead, the groups should only differ in their post-manipulation expectations. Accordingly, it appears necessary to replicate the experiment to see whether this unexpected main effect of condition merely reflects a Type-I error.

Interestingly, some group differences in expectation update came up when considering only the negatively worded expectation items for exploratory purposes. In this case, participants from the cognitive immunisation promotion condition updated their expectations to a lesser extent in a negative direction than participants from the cognitive immunisation inhibition condition and the control group, reflecting medium effects (of note, this effect was significant only for task-specific expectations). This suggests that the valence of expectation measures might matter, in the sense that the effects of a cognitive immunisation-promoting manipulation on expectation update are more pronounced for prior expectations with negative valence (e.g., the expectation of failure in a given task). Speculatively, this might be explained by the effect that defiance to unexpectedly negative feedback is greater if the wording of expectations suggests that one might have difficulty working on the tasks from the test; conceivably, the endorsement of such statements after receiving negative feedback is particularly weak if participants were informed previously that the test is fairly unreliable (as in the cognitive immunisation promotion group). Yet, as this analysis was exploratory only and not pre-planned, the findings need to be interpreted with caution and require replication.

Additional analyses examined depressive symptoms and dispositional optimism as predictors of expectation update. A linear regression analysis indicated that depressive symptoms were descriptively associated with a somewhat greater adjustment of expectations in line with the negative feedback received, but this effect did not reach significance. Dispositional optimism did not predict expectation update either.

In sum, this experiment demonstrated that although the experimental groups differed in their engagement in cognitive immunisation as expected, the modulation of cognitive immunisation did not significantly influence expectation adjustment, with the exception of including only the negatively worded expectations. To examine the robustness of these results, we aimed to replicate the experiment in another independent sample. That was the subject of Experiment 2.

## Experiment 2

### Methods

#### Participants

As in Study 1, participants were recruited via email lists and social networks. For this online experiment, participants were sent the link to the study and worked through the tasks on their own. Participants had the opportunity to contact the study team in case of any questions. The inclusion and exclusion criteria were the same as in Experiment 1.

### Procedure, Measures, and Statistical Analyses

The procedure of Experiment 2 was the same as for Experiment 1, except for the fact that it was conducted as an online experiment, since a laboratory experiment with physical contact was no longer possible due to the restrictions related to the Covid-19 pandemic. Data were collected between March and June 2020. In terms of the measures used and the statistical analyses performed, Experiment 2 did not differ from Experiment 1, which is why we do not reiterate their description here.

## Results

### Sample Characteristics

At total of 109 people participated in the online survey, and 93 individuals completed the study (*n* = 31 in the immunisation-inhibition group; *n* = 30 in the immunisation-promotion group; *n* = 32 in the control group). Of those people who completed the study, 76.3% were female and the mean age was *M* = 28.17 (*SD* = 11.88; range 18–65). A majority (63.5%) had a high school degree, 31.2% had a university degree, and 5.4% had primary education as the highest educational degree. Most participants (71.0%) were students; 15.1% were working full-time; 8.6% were working part-time; two participants (2.2%) were disabled; one participant (1.1%) was unemployed and another one was homemaker. On average, participants reported minimal levels of depression (*M* = 10.08; *SD* = 10.71); 17.4% of the sample reported elevated levels of depressive symptoms (BDI-II ≥ 19).

### Baseline Differences

Participants from the three groups did not differ in their initial task-specific expectations, *F* (2, 90) = 1.349, *p* = 0.265, *ɳ*^2^_*p*_ = 0.029, 95% CI [0, 0.104], initial generalised expectations, *F* (2, 90) = 1.282, *p* = 0.282, *ɳ*^2^_*p*_ = 0.028, 95% CI [0, 0.102], and age, *F*(2, 90) = 0.181, *p* = 0.835, *ɳ*^2^_*p*_ = 0.004, 95% CI [0, 0.040]. The distribution of male and female participants was not significantly different across the groups, *χ*^2^ = 2.620, *p* = 0.270, nor was the distribution of educational levels, *χ*^2^ = 14.784, *p* = 0.140, and employment status, *χ*^2^ = 9.118, *p* = 0.693.

### Manipulation Check

The one-way ANOVA indicated no significant differences between the groups in their CIPF total scores, *F* (2, 90) = 0.791, *p* = 0.457, *ɳ*^2^_*p*_ = 0.017, 95% CI [0, 0.086]. That is, the manipulation failed to differentially manipulate participants’ engagement in cognitive immunisation strategies.

### Main Analyses

#### Changes in Generalised Expectations

The Time by Condition two-factorial ANOVA with generalised expectations as the dependent variable indicated a significant main effect of Time, *F* (1, 90) = 4.653, *p* = 0.034, *ɳ*^2^_*p*_ = 0.049, 95% CI [0.001, 0.157]. Overall, participants lowered their expectations from pre (*M* = 10.08, *SD* = 2.56) to post (*M* = 9.53, *SD* = 2.53). The main effect of condition was not significant, *F* (2, 90) = 1.604, *p* = 0.207, *ɳ*^2^_*p*_ = 0.034, 95% CI [0, 0.120]. The Time by Condition interaction was not significant either, *F*(2, 90) = 0.012, *p* = 0.988, *ɳ*^2^_*p*_ < 0.001, 95% CI [0, 0.005].

#### Changes in Task-specific Expectations

The Time by Condition two-factorial ANOVA with task-specific expectations as the dependent variable indicated a significant main effect of Time, *F* (1, 90) = 57.588, *p* < 0.001, *ɳ*^2^_*p*_ = 0.390, 95% CI [0.235, 0.512]. Overall, participants lowered their expectations from pre (*M* = 10.75, *SD* = 2.31) to post (*M* = 8.15, *SD* = 2.72). The main effect of condition was not significant, *F* (2, 90) = 1.903, *p* = 0.155, *ɳ*^2^_*p*_ = 0.041, 95% CI [0, 0.130], nor was the Time by Condition interaction, *F* (2, 90) = 0.591, *p* = 0.556, *ɳ*^2^_*p*_ = 0.013, 95% CI [0, 0.075].

#### Considering Negatively Worded Expectations Only

As in Experiment 1, we examined whether the pattern of results changes if only negatively worded expectation items are included. For generalised expectations as the dependent variable, the main effect of time was not significant any longer in this analysis, *F* (1, 90) = 0.001, *p* = 0.980, *ɳ*^2^_*p*_ < 0.001, 95% CI [0, 0.001]. The main effect of condition was still non-significant, *F* (2, 90) = 2.302, *p* = 0.106, *ɳ*^2^_*p*_ = 0.049, 95% CI [0, 0.144]. While a non-significant trend (*p* = 0.056) was found in Experiment 1 for the Time by Condition interaction, this interaction was far from being significant in Experiment 2, *F* (2, 90) = 1.105, *p* = 0.336, *ɳ*^2^_*p*_ = 0.024, 95% CI [0, 0.100]. When considering the negatively worded task-specific expectations scale as the dependent variable, the main effect of Time was significant, *F* (1, 90) = 8.866, *p* = 0.004, *ɳ*^2^_*p*_ = 0.090, 95% CI [0.010, 0.212], whereas the main effect of condition was not, *F* (2, 90) = 1.741, *p* = 0.181, *ɳ*^2^_*p*_ = 0.037, 95% CI [0, 0.125]. Unlike Experiment 1, Experiment 2 indicated no significant Time by Condition interaction, *F* (2, 90) = 1.707, *p* = 0.187, *ɳ*^2^_*p*_ = 0.037, 95% CI [0, 0.124].

### Depressive Symptoms and Optimism as Predictors of Expectation Update

The regression analysis including depressive symptoms and dispositional optimism as predictors of pre to post changes in generalised performance expectations was performed as described for Experiment 1. The results of this regression analysis indicated that the experimental condition did not have significant effects (*R*^2^ = 0.001; *β* = 0.011; *p* = 0.914). Entering depressive symptoms as a predictor in the second block explained 1.4% of the variance in expectation update, which was not significant (*β* = 0.118; *p* = 0.264). Adding LOT-R sum scores as predictors in the second block did not significantly add explained variance (*ΔR*^2^ = 0.026; *β* = 0.209; *p* = 0.124).

## Discussion

Similar to Experiment 1, this experiment failed to detect significant differences between the groups in updating their performance-related expectations. That is, the cognitive immunisation manipulation did not affect the adjustment of expectations in response to unexpectedly negative feedback. Rather, participants from all experimental groups updated their expectations in line with the feedback received (when considering the entire expectation scale). As with Study 1, it remains unclear, though, how stable these changes in expectations were due to the lack of a follow-up assessment some time later. Unlike Study 1, Study 2 failed to produce significant group differences in cognitive immunisation; that is, it may be that the lack of group differences in expectation updating is due to the ineffectiveness of the cognitive immunisation manipulation. A further difference between the results of the two experiments is that while Experiment 1 found that group differences in expectation updating were more pronounced when considering only negatively worded expectations, this effect was not replicated in Experiment 2, neither for generalised nor for task-specific expectations. Thus, the trend found in Experiment 1 did not prove to be robust in Experiment 2.

With regard to depressive symptoms, Experiment 2—like Experiment 1—did not find any significant association of depressive symptoms on belief updating in response to negative feedback, as examined in a linear regression analysis. These findings suggest that depression is not related to an increased sensitivity to negative feedback, as supported by other research (Brolsma et al., 2020; Kube et al., 2019a, b, c). Similar to Experiment 1, Experiment 2 did not find any association between dispositional optimism and expectation adjustment.

## Experiment 3

In contrast to Experiment 1 & Experiment 2, this experiment examined the update of expectations in response to unexpectedly positive information. Moreover, we investigated possible temporal changes in expectation update by adding a 2-week follow-up. That is, we examined whether participants’ adjustment of expectations in response to feedback persisted 2 weeks later or whether it diminished over time. This follow-up analysis will be informative in regard to the temporal stability of belief updating, as a recent study has shown that the preferred integration of desirable feedback as compared to undesirable feedback becomes even more pronounced over time (Yao et al., 2021).

### Participants

As in the other experiments, participants were recruited via email lists and social networks. Participants were sent the link to the online study and worked through the tasks on their own. The inclusion and exclusion criteria were the same as in Experiment 1 & Experiment 2.

### Procedure and Experimental Groups

The procedure of Experiment 3 was the same as for the other two experiments, except for the fact that participants in this study received unexpectedly positive feedback. In particular, they received the feedback that they were among the best 15% of all participants who worked on the TEMINT. To make sure that this feedback appears unexpectedly positive, participants’ initial expectations for their performance were lowered. To this end, they were informed at the very beginning of the study that they were about to take a very difficult test that hardly anyone can solve correctly, without being aware of which particular test they would have to work on. In particular, participants received the following instruction: “*Up to now, you should not be familiar with the tasks from the test. The tasks were designed by the developers to be very difficult and to be solved correctly by only a few people.*” This procedure of lowering participants’ baseline expectations and providing them with surprisingly positive feedback afterwards has been shown to be appropriate in examining intra-individual changes in performance-related expectations (Kube et al., 2018). Previous research indicated that healthy people adjust their expectations in line with that positive feedback, whereas people with depression maintain their initial expectations (Kube et al., 2019c).

To examine how a modulation of cognitive immunisation influences expectation adjustment, participants were randomised to a cognitive immunisation-promoting vs.—inhibiting condition or a control condition. To this end, they received the same information texts as used for Experiment 1 & Experiment 2.

Data were collected online between December 2020 and February 2021. In terms of the measures used, Experiment 3 did not differ from Experiment 1 & Experiment 2, which is why we do not reiterate their description here. To match participants’ data from the first assessment with the follow-up assessment 2 weeks later, they were asked to generate a personal code, comprised of the first two letters of their place of birth, the first two letters of their first name, and the month they were born in (e.g., BeTo08).

### Statistical Analyses

The basic procedure for the statistical analyses being performed was the same as for the previous experiments. However, as Experiment 3 comprised an additional assessment 2 weeks later, the main analysis was a 3 (Time: before feedback (pre) vs. shortly after feedback (post) vs. 2 weeks later (follow-up)) × 3 (Condition: immunisation inhibition vs. immunisation promotion vs. control group) × 2 mixed ANOVA, with the generalised performance expectations as the dependent variable. Moreover, as in the previous experiments, we performed a linear regression analysis to examine the effects of depression and optimism on expectation change.

## Results

### Sample Characteristics

At total of 138 people participated in the study, of whom 118 completed the experiment at the first assessment day and endorsed the control items correctly. Of these, 105 completed the follow-up assessment 2 weeks later. Unfortunately, we could match the data of only 88 people from the follow-up with their data from the first assessment; for the other 17 people, the personal code entered at the follow-up did not correspond to any of the codes generated 2 weeks earlier (although the information requested for the generation of the code in fact should not have changed). Thus, the analyses were based on data from 118 people for the first assessment (with n = 39 for the immunisation-promotion condition, n = 39 for the immunisation-inhibition condition, and n = 40 for the control condition) and 88 people for the follow-up (with n = 29 for the immunisation-promotion condition, n = 28 for the immunisation-inhibition condition, and n = 31 for the control condition).

Participants’ mean age was 26.68 years (*SD* = 5.52). In our sample, 80.7% of the participants were female, and 75.0% were undergraduate students (most of which studied psychology). Sociodemographic characteristics for the three experimental groups separately can be found in Table 3. BDI-II sum scores (*M* = 11.65; *SD* = 8.19) indicate that on average participants reported minimal levels of depressive symptoms. Seven participants (7.1%) reported elevated levels of depression (BDI-II sum scores ≥ 19).

**Table 3:** Sample characteristics in study 3

Variable	Immunisation-inhibiting group (*n* = 28)	Immunisation-promoting group (*n* = 29)	Control group (*n* = 31)
Age in years, *M* (SD)	25.50 (10.41)	28.90 (14.10)	25.68 (9.38)
Sex, N (%)			
Male	5 (17.9)	5 (17.2)	7 (22.6)
Female	23 (82.1)	24 (82.8)	24 (77.4)
Educational level, N (%)			
High school degree	24 (85.7)	20 (69.0)	26 (83.9)
University degree	4 (14.3)	7 (24.1)	5 (16.1)
Employment status, N (%)			
Full-time working	4 (14.3)	6 (20.7)	4 (12.9)
Part-time working	2 (7.1)	2 (6.9)	2 (6.5)
In training	22 (78.6)	19 (65.5)	25 (80.6)
Unemployed	0	1 (3.4)	0

*M* mean, *SD* standard deviation, *N* number

### Baseline Differences

Participants from the three groups did not differ in their initial task-specific expectations, *F* (2, 115) = 2.052, *p* = 0.133, *ɳ*^2^_*p*_ = 0.034, 95% CI [0, 0.110], initial generalised expectations, *F* (2, 115) = 0.404, *p* = 0.669, *ɳ*^2^_*p*_ = 0.007, 95% CI [0, 0.051], and age, *F* (2, 115) = 1.039, *p* = 0.357, *ɳ*^2^_*p*_ = 0.018, 95% CI [0, 0.079]. The distribution of male and female participants was not significantly different across the groups, *χ*^2^ = 1.994, *p* = 0.369, nor was the distribution of educational level, *χ*^2^ = 11.244, *p* = 0.188, and employment status, *χ*^2^ = 6.113, *p* = 0.635.

### Manipulation Check

The one-way ANOVA indicated significant differences between the groups in their CIPF total scores, *F* (2, 115) = 4.431, *p* = 0.014, *ɳ*^2^_*p*_ = 0.072, 95% CI [0.003, 0.166]. Pairwise comparisons showed that the immunisation-inhibiting condition had significantly lower CIPF total scores (*M* = 18.82; *SD* = 6.04) than the immunisation-promoting condition (*M* = 23.08; *SD* = 6.74), *t* (76) = 2.934; *p* = 0.004; *d* = 0.665, 95% CI [0.241, 1.309] and the control group (*M* = 22.60; *SD* = 7.84), *t* (77) = -2.394; *p* = 0.019; *d* = 0.540, 95% CI [0.135, 1.136], reflecting medium effects according to Cohen (1988). The immunisation-promoting condition and the control group did not differ significantly in their CIPF total scores, *t* (77) = 0.289; *p* = 0.773; *d* = 0.065, 95% CI [−0.432, 0.581]. This indicates that the manipulation was successful in differentially manipulating participants’ appraisal of the feedback received. It should be noted, though, that the manipulation was only successful in reducing the engagement in cognitive immunisation in the immunisation-inhibition group, but not in increasing it in the immunisation-promotion group.

### Main Analyses

#### Changes in Generalised Expectations

Considering the measurements before and after feedback, the Time by Condition ANOVA indicated a significant main effect of Time, *F* (1, 115) = 54.856, *p* < 0.001, *ɳ*^2^_*p*_ = 0.323, 95% CI [0.189, 0.439], with more positive generalised performance expectations after feedback than before (see Table 4). The main effect of Condition was not significant, *F* (2, 115) = 0.367, *p* = 0.694, *ɳ*^2^_*p*_ = 0.006, 95% CI [0, 0.048]. The Time by Condition interaction was not significant either, *F* (2, 115) = 0.151, *p* = 0.860, *ɳ*^2^_*p*_ = 0.003, 95% CI [0, 0.031].^3^

**Table 4:** Descriptive values for the expectation ratings in study 3

Variable	Immunisation-inhibiting group (*n* = 28)	Immunisation-promoting group (*n* = 29)	Control group (*n* = 31)
Task-specific expectations, *M* (*SD*)			
Pre	7.96 (2.85)	8.10 (2.78)	9.03 (2.93)
Post	12.00 (1.33)	11.72 (1.69)	11.77 (1.67)
Follow-up	11.50 (1.55)	10.79 (1.72)	10.58 (1.91)
Generalised expectations, *M* (*SD*)			
Pre	8.50 (2.49)	9.17 (2.64)	9.06 (2.86)
Post	10.18 (2.88)	10.34 (2.38)	10.48 (2.49)
Follow-up	10.29 (2.14)	10.07 (1.89)	10.00 (2.11)

*M* mean, *SD* standard deviation

**Fig. 4:**
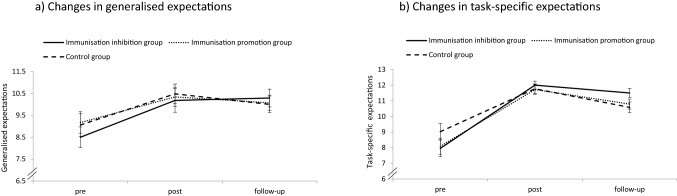
Results for the main analyses from Experiment 3. There were no significant differences between the groups in updating their expectations from pre to post to follow-up. Error bars reflect the standard error or the mean

Including the follow-up assessment as an additional measurement, the Time by Condition ANOVA indicated a significant main effect of Time, *F* (2, 85) = 21.324, *p* < 0.001, *ɳ*^2^_*p*_ = 0.201, 95% CI [0.168, 0.458], with overall lower expectations before feedback than shortly after feedback and at follow-up (see Table 4 for the descriptive values). The main effect of condition was not significant, *F* (2, 85) = 0.088, *p* = 0.916, *ɳ*^2^_*p*_ = 0.0024, 95% CI [0, 0.040]. The Time by Condition interaction was not significant either, *F* (4, 85) = 0.803, *p* = 0.525, *ɳ*^2^_*p*_ = 0.019, 95% CI [0, 0.098]. Thus, participants from all groups significantly updated their expectations in line with the positive feedback received, and this effect remained stable 2 weeks later. The results of this analysis are depicted in Fig. 4.

#### Changes in Task-specific Expectations

Considering the measurements before and after feedback, the Time by Condition ANOVA indicated a significant main effect of Time, *F* (1, 115) = 132.638, *p* < 0.001, *ɳ*^2^_*p*_ = 0.536, 95% CI [0.411, 0.625], with more positive task-specific expectations after feedback than before (see Table 4). The main effect of Condition was not significant, *F* (2, 115) = 2.399, *p* = 0.095, *ɳ*^2^_*p*_ = 0.040, 95% CI [0, 0.119], nor was the Time by Condition interaction, *F* (2, 112) = 0.858, *p* = 0.427, *ɳ*^2^_*p*_ = 0.015, 95% CI [0, 0.072].

Including the follow-up assessment as an additional measurement, the Time by Condition ANOVA indicated a significant main effect of Time, *F* (1.363, 115.691) = 76.597, *p* < 0.001, *ɳ*^2^_*p*_ = 0.474, 95% CI [0.341, 0.572]. Overall, participants’ expectations were significantly higher after receiving feedback than before feedback, *t* (87) = -−0.510; *p* < 0.001; *d* = 1.579, 95% CI [0.851, 1.384], and participants’ expectations declined somewhat from shortly after feedback to follow-up, *t* (87) = 5.341; *p* < 0.001; *d* = −0.610, 95% CI [0.444, 0.905], although participants’ expectations at follow-up were still significantly higher than at baseline, *t* (87) = −7.222; *p* < 0.001; *d* = 0.640, 95% CI [0.531, 1.005]. That is, a significant change in participants’ expectations remained stable over 2 weeks, although the degree of change became somewhat smaller as compared to shortly after receiving feedback. The descriptive values are presented in Table 4. The main effect of condition was not significant, *F* (2, 85) = 0.339, *p* = 0.713, *ɳ*^2^_*p*_ = 0.008, 95% CI [0, 0.061], nor was the Time by Condition interaction, *F* (2.722, 115.691) = 2.058, *p* = 0.116, *ɳ*^2^_*p*_ = 0.046, 95% CI [0, 0.122].

Of note, the pattern of results did not change significantly when examining only the negatively worded items, which is why we do not present this extra analysis here in detail again.

### Depressive Symptoms and Optimism as Predictors of Expectation Update

The regression analysis including depressive symptoms and dispositional optimism as predictors of in generalised performance expectations from baseline to follow-up was performed as described for Experiment 1 & Experiment 2. The results of this regression analysis indicated that the experimental condition had no significant effects, *R*^2^ = 0.001; *β* = 0.004; *p* = 0.973. Entering depressive symptoms as a predictor in the second block explained an additional 4.7% of the variance in expectation update, which was significant (*β* = −0.220; *p* = 0.043), indicating that depressive symptoms were related to a reduced update in line with the positive feedback received. Adding the LOT-R sum score as a predictor in the third block did not significantly add explained variance (*ΔR*^*2*^ = 0.007; *β* = 0.101; *p* = 0.418).

## Discussion

In this experiment, we investigated whether a cognitive immunisation manipulation leads to differences in updating performance-related expectations in response to unexpectedly positive performance feedback. In a 2-week follow-up, we also examined how stable such changes in participants’ expectations are. We found that although the cognitive immunisation manipulation was effective in differentially manipulating participants’ appraisal of the feedback received, the manipulation had no influence on the adjustment of expectations. Rather, participants from all experimental groups updated their expectations in line with the positive feedback received. For generalised expectations, this positive update remained stable over 2 weeks, while the significant update of task-specific expectations slightly declined from immediately after feedback to 2 weeks later. The former appears remarkable as it suggests that receiving unexpectedly positive performance feedback in a previously unknown task entails a relatively stable adjustment of participants’ general expectation of their own abilities. An additional finding of this experiment was that depressive symptoms were associated with reduced expectation updating in line with the positive feedback received, while dispositional optimism had no incremental effects on updating.

## General Discussion

The aim of the present series of experiments was to investigate how cognitive immunisation against new information influences the adjustment of expectations in three non-clinical samples. To this end, we provided participants with unexpectedly negative (see section “Experiment 1”& “Experiment 2”) and positive (see section “Experiment 3”) performance feedback and examined how a modulation of the appraisal of the feedback affected the extent to which initial performance expectations were changed. We found that the manipulation of cognitive immunisation had no significant effect on the adjustment of expectations in response to feedback in any of the three experiments. Rather, we found that participants from all experimental groups updated their expectations in line with the feedback received, regardless of a manipulation aimed at increasing vs. lowering the value of the feedback. In Experiment 3, we found that changes in expectations were maintained over 2 weeks.

To interpret these findings, it is important to consider the results of the manipulation checks. Though the cognitive immunisation manipulation was successful in differentially modulating the appraisal of the feedback received in Experiment 1 and Experiment 3, it had no significant effect in Experiment 2, and even the effects in Experiment 1 and Experiment 3 were of rather moderate size. This implies that the lack of group differences in expectation updating might be related to the fact that the cognitive immunisation manipulation was not strong enough to produce larger effects. Another interpretation is that the feedback itself had such a strong influence on the adjustment of expectations that any kind of manipulation could not have a significant effect over the feedback. Relatedly, it is possible that the statistical power of each experiment was not high enough to uncover small effects of cognitive immunisation on expectation change. This interpretation would be consistent with the descriptive trends found in Experiment 1 and Experiment 3, which pointed to group differences in the hypothesised direction. The power calculation was based on the results of previous studies using clinical samples, where the effects of cognitive immunisation manipulations on expectation change were found to be larger (Kube et al., 2019a and c). Thus, it seems that while cognitive immunisation is critically involved in belief updating in clinically depressed people, as shown previously, the effects in non-clinical samples are considerably lower or even non-existent.

Relatedly, it might be that the specific cognitive strategy by which disconfirming evidence is devalued differs between clinical and non-clinical samples: It is possible that while people with depression are prone to assigning little reliability to disconfirming (positive) information, healthy people engage more in concept-oriented cognitive immunisation strategies (Panitz et al., 2021). Since the cognitive immunisation manipulation of the present studies was tied to the increase vs. decrease of the value of disconfirming information only, future research may investigate whether the promotion vs. inhibition of other types of cognitive immunisation strategies have stronger effects on the extent to which beliefs are updated.

The present findings add to previous research into belief updating in mental health vs. disorders (Kube & Rozenkrantz, 2021). In previous work, it has been shown that people with depression have difficulty updating negative beliefs after receiving novel positive information (Everaert et al., 2018, 2020; Korn et al., 2014; Liknaitzky et al., 2017). Our findings are consistent with that work as the results of Study 3 indicated that depressive symptoms were related to a reduced expectation update in line with unexpectedly positive feedback. In contrast, we did not find any significant effects of depressive symptoms on belief updating in response to negative feedback, as examined in Study 1 and Study 2. This is consistent with previous research suggesting that depression is related to a reduced integration of novel positive information, rather than to an increased sensitivity to unexpectedly negative information (Brolsma et al., 2020; Everaert et al., 2018; Kube et al., 2019b). Of note, given the small/null effects of the cognitive immunisation manipulation on belief updating, we decided against performing a moderation analysis to see whether the effects of condition interact with depression, although it would be of theoretical interest to examine such an interaction.

In another respect, the results of Experiment 1 & Experiment 2 are to some extent at odds with the optimism bias/positivity bias which has previously been associated with mental health: Whereas previous research has shown that healthy people exhibit little update of their beliefs in response to bad news—both in the context of new information on the self (Korn et al., 2012) and new factual information (Garrett & Sharot, 2017; Sharot et al., 2011)—we did find that participants lowered their expectations about their performance after receiving negative feedback in Experiment 1 and Experiment 2. A possible interpretation of this discrepancy is that performance feedback could be processed differently than feedback on personality traits, as examined in the study by Korn et al., (2012), with negative performance feedback having more influence on belief updating than negative feedback about one’s personality. This interpretation would be consistent with results from social and personality psychology on self-concept stability, suggesting that (healthy) people tend to reject negative feedback that is inconsistent with their self-concept (Markus, 1977; Swann & Read, 1981).

Interestingly, the results of Experiment 3 suggest that the adjustment of beliefs about the self in response to positive performance feedback are relatively stable, as indicated by the sustained changes in expectations at the 2-weeks follow-up. This is remarkable as it suggests that in healthy people, a single positive experience that disconfirms previous negative expectations is sufficient to elicit a sustained update of expectations. For people with depression, on the other hand, it has been assumed that a considerable number of disconfirming positive experiences is needed to revise established negative expectations (Kube et al., 2020; Rief & Joormann, 2019).

Of note, none of the three experiments reported here found any predictive value of dispositional optimism in relation to belief updating. This is in accordance with other research including optimism as a continuous variable (Kube & Glombiewski, 2021; Kube et al., 2019c), whereas significant effects of trait optimism on belief updating were only found if it was dichotomised, i.e. high vs. low optimism (Sharot et al., 2011). Hence, the majority of findings point to the interpretation that dispositional optimism in the sense of a relatively stable personality trait is not involved in updating beliefs about the self. The fact that Sharot et al., (2011) did find significant effects of trait optimism on belief updating might be related to their particular statistical approach (which may be criticised from a statistical point of view since the artificial dichotomisation of variables is related to a loss of information). Alternatively, it is possible that the discrepancy in the results is related to the different task Sharot et al. used: Their belief-updating task was tied to the expectation of adverse future life events, and it might be that dispositional optimism in terms of a generalised positive outcome expectation is involved in that task, rather than in the present task with a narrower focus on performance.

### Strengths and Limitations

To our knowledge, our work is the first to systematically investigate the influence of cognitive immunisation on belief updating in non-clinical samples, both in response to unexpectedly positive and negative feedback. Further strengths can be seen in the use of an experimental modulation protocol that was added to previously validated experimental paradigms, allowing to test some causal effects (Jacoby & Sassenberg, 2011; Lemmer & Gollwitzer, 2017), and its application in three consecutive experiments, the second of which was designed to replicate the results of the first experiment. Furthermore, by adding a 2-week follow-up, the third experiment was the first to examine the temporal stability of the adjustment of expectations in response to disconfirming feedback. Notwithstanding these merits, the present studies also have limitations that need to be considered.

A general limitation applying to all three experiments is that the studies were based on self-report measures only, leaving some potential of demand effects and limiting the robustness of the findings. Furthermore, both the expectation scales and the CIPF consist of a small number of items, thus lowering the reliability of these measures, which may have contributed to the failure to find significant effects of the cognitive immunisation manipulation on expectation updating. Another general limitation is that we focused on expectations for performance only and did not consider other types of expectations that might be important in the context of expectation updating as well, such as expectations for social rejection (D'Astolfo et al., 2020) or expectations for future life events (Sharot et al., 2011). Moreover, the likelihood of finding significant effects of depression was limited due to the low number of people reporting elevated levels of depression in all experiments. Another limitation is that we do not know for sure how the control group in each experiment appraised the feedback received, while the other two conditions received specific instructions in this regard. In addition, we could not statistically control for the potential effects of practice with the TEMINT on changes in expectations. A further limitation pertaining to the first two experiments is that Experiment 2 failed to replicate the success of the cognitive immunisation manipulation of Experiment 1, in terms of eliciting differential engagement in cognitive immunisation strategies in the three experimental groups. We suggest that the failure to replicate the successful manipulation check might be attributed to the fact that Experiment 2 was performed as an online experiment, whereas Experiment 1 was conducted in the laboratory. We guess so, because aside from that issue, the two experiments did not differ in any other respect. Alternatively, it is also possible that the significant differences in cognitive immunisation as found in Experiment 1 were chance findings, and in fact cognitive immunisation manipulations do not work in non-clinical samples, but only in clinically depressed people (Kube et al., 2019a and c).

## Conclusion

In a series of three experiments, we investigated how cognitive immunisation against disconfirming evidence contributes to belief updating in non-clinical samples. We found that differences in the appraisal of disconfirming evidence did not influence the adjustment of expectations, in contrast to previous studies examining people with depression. Consistent with previous work, we found that depressive symptoms—even in a non-clinical sample—were related to a reduced integration of positive performance feedback. In sum, the current findings suggest that non-clinical samples clearly update their beliefs about their performance when provided with disconfirming feedback, regardless of cognitive immunisation manipulations.

## Supplementary Information

Below is the link to the electronic supplementary material.

Supplementary file1 (DOCX 17 kb)

## Footnotes

1 Entering the predictors in a different order did not change their effects on the results.

2 As indicated in the pre-registration of the study, we also ran the main analysis of the study (that is, the two-factorial ANOVA) without the seven participants reporting elevated levels of depression. This analysis is reported in the Supplement. As can be seen there, the pattern of results did not significantly change when excluding these participants.

3 Due to sex imbalance, we re-ran the analyses only among female participants. This did not significantly change the results; that is, it had no influence on the significance of any of the effects, nor did it change the effect sizes substantially.

## Declarations

### Research Involving Human and Animal Participants

No animal studies were carried out by the authors for this article.
