# Supplementary material for: No Evidence for the Involvement of Cognitive Immunisation in Updating Beliefs About the Self in Three Non-Clinical Samples
Source: Cognit Ther Res. 2021 Jul 30;46(1):43–61. doi: 10.1007/s10608-021-10256-y (PMC8323093; doi:10.1007/s10608-021-10256-y)
Supplement: Supplementary file 1 — Supplementary file1 (DOCX 17 kb) [file 10608_2021_10256_MOESM1_ESM.docx]

**Supplementary Material**

# Main analyses from Experiment 1 without participants reporting elevated symptoms of depression

***Changes in generalised expectations*.** The Time by Condition two-factorial ANOVA with generalised expectations as the dependent variable indicated a significant main effect of Time, *F*(1, 89) = 14.341, *p* < .001, *ɳ²_p_* = .139. Overall, participants lowered their expectations from pre (*M* = 9.76, *SD* = 2.12) to post (*M* = 8.89, *SD* = 2.38). The main effect of condition was not significant, *F*(2, 89) = 2.223, *p* = .114, *ɳ²_p_* = .048. The Time by Condition interaction was not significant either, *F*(2, 89) = 0.658, *p* = .520, *ɳ²_p_* = .015.

***Changes in task-specific expectations*.** The Time by Condition two-factorial ANOVA with task-specific expectations as the dependent variable indicated a significant main effect of Time, *F*(1, 89) = 38.395, *p* < .001, *ɳ²_p_* = .301, with overall less positive expectations after feedback (*M* = 8.28, *SD* = 2.54) than before feedback (*M* = 10.29, *SD* = 2.05). The main effect of Condition was significant, *F*(2, 89) = 6.411, *p* = .003, *ɳ²_p_* = .126. Pairwise *t*-tests indicated that, overall, participants from the immunisation-promoting group (*M* = 20.13; *SD* = 3.46) reported significantly more positive expectations than participants from the immunisation-inhibiting group (*M* = 18.29; *SD* = 2.17), *t*(58) = -2.423; *p* = .019; *d* = .637, and the control group (*M* = 17.28; *SD* = 3.69), *t*(62) = 3.179; *p* = .002; *d* = .796, reflecting medium to large effects. The difference between the immunisation-inhibiting group and the control group was not significant, *t*(58) = 1.260; *p* = .213; *d* = .333. As for generalised expectations, the Time by Condition interaction was not significant, *F*(2, 89) = 1.236, *p* = .296, *ɳ²_p_* = .027.
